# Imaging for Quality Control: Comparison of Systematic Video Recording to the Operative Note in Colorectal Cancer Surgery. A Pilot Study

**DOI:** 10.1245/s10434-016-5563-y

**Published:** 2016-09-22

**Authors:** F. W. van de Graaf, M. M. Lange, A. G. Menon, P. R. A. O’Mahoney, J. W. Milsom, J. F. Lange

**Affiliations:** 1 Department of Surgery, Erasmus University Medical Center, Rotterdam, The Netherlands; 2 Department of Surgery, Leiden University Medical Center, Leiden, The Netherlands; 3 Department of Surgery, Havenziekenhuis, Rotterdam, The Netherlands; 4 Section of Colon and Rectal Surgery, Department of Surgery, NewYork-Presbyterian Hospital, Weill Cornell Medical College, New York, NY USA

## Abstract

**Background:**

Oncological and functional results after colorectal cancer surgery vary considerably between hospitals and surgeons. At present, the only source of technical information about the surgical procedure is the operative note, which is subjective and omits critical information. This study aimed to evaluate the feasibility of operative video recording in demonstrating both objective information concerning the surgical procedure and surgical quality, as using a systematic approach might improve surgical performance.

**Methods:**

From July 2015 through November 2015, patients aged ≥18 years undergoing elective colorectal cancer surgery were prospectively included in a single-institution trial. Video recording of key moments was performed peroperatively and analyzed for adequacy. The study cases were compared with a historic cohort. Video was compared with the operative note using the amount of adequate steps and a scoring system.

**Results:**

This study compared 15 cases to 32 cases from the historic control group. Compared to the written operative note alone, significant differences in availability of information were seen in favor of video as well as using a combination of video plus the operative note (N adequate steps *p* = .024; *p* = <.001. Adequacy score: *p* = .039; *p* = <.001, both respectively).

**Conclusions:**

Systematic video registration is feasible and seems to improve the availability of essential information after colorectal cancer surgery. In this respect, combining video with a traditional operative note would be the best option. A multicenter international study is being organized to further evaluate the effect of operative video capture on surgical outcomes.

Over the past several years, laparoscopic surgery has become standard of care in the treatment of colorectal malignancies, resulting in similar oncological outcomes and improved short-term results compared with conventional open surgery.^1^,^2^

Although colorectal cancer treatment has improved dramatically, short- and long-term oncological and functional results in colorectal cancer patients with similar stage disease vary widely between different hospitals and surgeons.^3^ Operative mortality in colorectal cancer patients ranges from 0.5 to 6 %, while operative morbidity ranges from 15 to 25 %, mainly as a result of avoidable surgical complications.^1^,^4^–^6^ Regarding oncological outcome, disease recurrence is reported in 5–50 % of patients and 5-year survival rates vary between 32 and 64 %.^7^–^10^ Long-term pelvic organ dysfunction after rectal cancer surgery, mainly attributed to avoidable surgical (nerve) damage, occurs in the majority of patients.^10^–^14^ In this respect surgical performance in colorectal cancer surgery still has room for improvement, especially with regard to reducing variability among surgeons.

The importance of quality improvement programs to decrease operative variability is widely supported at this time. In 2009, Haynes et al. introduced the surgical safety checklist, used in the “Time-out-procedure,” cutting mortality in half after implementation.^15^ However, this checklist addresses only preoperative anesthesiological and nursing concerns and not so much the surgical technique used during surgery. Furthermore, surgical quality is an important prognostic factor, especially in colorectal cancer treatment, but is poorly captured. During complex surgical procedures, such as total mesorectal excision (TME) for rectal cancer, essential steps might be skipped or inadequately performed (such as the identification of nerves and the ureter). However, postoperatively, it cannot be clearly reproduced what exactly occurred during the surgical procedure. Currently, the only source of technical information about the surgical procedure is the operative note, which has been shown to be subjective and lacking in critical information.^16^ Systematic video registration of the procedure might be a solution, adding objective information to the traditional operative note.

The aim of this study was to investigate the feasibility of operative video recording with the hypothesis that this may (1) increase the amount of critical information from the surgical procedure and (2) improve surgical quality due to a systematic approach using a checklist.

## Methods

All patients aged 18 years or older undergoing elective laparoscopic colorectal cancer resection (right hemicolectomy, transverse colectomy, left hemicolectomy, sigmoid colectomy, or anterior resection) in Havenziekenhuis (Rotterdam, The Netherlands) were included from July 2015 through November 2015. During each surgical procedure, intraoperative video recordings of about 10 s length were made of predefined key moments, initiated and ceased by the primary surgeon. Patients with metastatic disease, unresectable tumor, or incomplete video recordings due to technical difficulties in the recording software were excluded from analysis. The primary and secondary outcomes were compared with a historical cohort, treated 1 year before implementation of the checklist, to avoid bias induced by the use of the checklist. The medical research and ethics committee of the Erasmus Medical Centre exempted this study from the Research Involving Human Subjects Act (WMO).

### Predefined Checklist and Reviewing

Key moments in the studied surgical procedures were defined by experts in this field (J.M., J.L., M.L.). A surgical checklist was compiled from these key moments and further transcribed into a case report form (CRF). During surgery, video fragments under direction of the leading surgeon were recorded according to the surgical checklist, and the corresponding steps were checked off on the CRF afterward. If a step was not relevant in a particular procedure, “n/a” was added next to the step in the CRF.

Before reviewing the video recordings, requirements for adequacy were dictated (F.vdG., J.M., A.M., J.L.). Then, the completed CRFs along with operative recordings and the operative notes were reviewed for adequacy (F.vdG., M.L.). The CRFs and the requirements for an adequate recording can be found in the online supplementary material. Failure to comply with these requirements, or absence of a recording resulted in a step being labeled “not adequate.” In reviewing the operative note, a step would be labeled “not adequate” if there were either an incomplete description or a lack of description altogether.

### Primary Outcome

With respect to the primary outcome, the availability of essential information, according to the predefined checklist, was evaluated. This information was collected from the operative video recording of the study group and from the operative note from both the study group and historic control group. Subsequently, the availability of the essential information was compared between the video recordings alone vs the operative note of the historic control group, and between the combination of the video recordings and the operative note coming from the study group vs the operative note of the historic control.

To assess the availability of information, two methods were used: (1) The adequacy of steps with adequate information was compared. (2) A scoring system was utilized. For the maximum amount of information, according to the critical steps described in the CRF, a maximum of 100 points could be obtained. These 100 points were divided by the number of applicable steps in that specific procedure, resulting in the amount of points per step. Finally, the factor of the amount of adequate steps and the amount of points per step was calculated, resulting in the total score for that specific procedure.

### Secondary Outcome

With respect to the secondary outcome, surgical complications within 30 postoperative days were analyzed to assess any improvement in surgical quality, which was expected with the use of a predefined checklist according to our hypothesis. Surgical complications were graded according to the Clavien-Dindo classification. The following complications were included in analysis: surgical wound infection, intra-abdominal abscess, urinary tract infection, respiratory tract infection, cardiologic complication, neurologic complication (including delirium), postoperative ileus, postoperative bleeding, and anastomotic leakage. Furthermore, the postoperative length of stay was measured.

### Video Recording and Video Data Management

High-definition images were captured using EndoEYE 30° videoscope connected to an EVIS EXERA II CLV-180 xenon light source and subsequently an EVIS EXERA II CV-180 video processor (Olympus Europa SE & Co., Hamburg, Germany). The video feed was then recorded on a Microsoft Windows based computer system in MPEG-4 format using image storage software (Clinical Assistant 6 [RVC Ltd., Baarn, The Netherlands]).

### Statistical Analysis

Data was analyzed with IBM SPSS version 23.0 for Mac. Categorical data were presented as numbers and percentages. Continuous data were described by mean and standard deviation. Study population and historical control were compared using chi-square test or Fisher exact test in case of categorical data. Continuous data was tested for normality using the Shapiro-Wilk Test, and if normal distribution was present, an independent samples *t* test was used. Otherwise, the Mann-Whitney *U* test was conducted. In analyzing the adequacy per step, the sum of all adequate steps was calculated, converting the range to continuous data. Subsequently the Mann-Whitney *U* test was used for comparison. A *p* value of less than .05 was considered statistically significant.

## Results

### Study Population

From July 2015 through November 2015, 20 patients meeting inclusion criteria were included in this study. All 20 patients underwent elective surgery for colorectal cancer with operative video recording according to the predefined checklist. A total of five patients were excluded from further analysis: two patients because of technical difficulties in the recording software resulting in loss of video fragments, two patients because of absence of malignancy, and one patient because of disseminated disease. As a result, the study population concerned 15 patients, who were was compared with 32 patients meeting inclusion criteria, which were retrospectively included in the period of July 2014 through January 2015. Patient characteristics are presented in Table 1. There were no significant demographic differences found between the study and the historic control group.

**Table 1:** Patient characteristics

Parameter	Study cases (*n* = 15)	Historic control (*n* = 32)	*p* value
Age (years)	67.87 ± 8.31	69.34 ± 14.16	.355
Sex			.758
Male	9 (60.0)	17 (53.1)	
Female	6 (40.0)	15 (49.6)	
Height (cm)	173.33 ± 11.60	171.94 ± 11.48	.654
Weight (kg)	85.69 ± 17.22	78.51 ± 15.98	.153
BMI (kg/m^2^)	28.49 ± 5.46	26.41 ± 4.13	.147
ASA class			.845
ASA I	1 (6.7)	4 (12.5)	
ASA II	6 (40.0)	18 (56.3)	
ASA III	2 (13. 3)	10 (31.3)	
Missing	6 (40.0)	0	
Charlson comorbidity index	2.79 ± 1.05	2.72 ± 0.99	.864
Diabetes mellitus	5 (33.3)	5 (15.6)	.242
Hypertension	8 (53.3)	20 (62.5)	.753
History of cardiac disease	2 (13.3)	7 (21.9)	.701
History of pulmonary disease	1 (6.7)	6 (18.8)	.413
History of renal disease	2 (13.3)	1 (3.1)	.216
Prior abdominal and/or pelvic surgery	6 (40.0)	13 (40.6)	1.000
Type of laparoscopic surgery			.369
Right hemicolectomy	3 (20.0)	11 (34.4)	
Transverse colectomy	1 (6.7)	0 (0)	
Left hemicolectomy	1 (6.7)	1 (3.1)	
Sigmoidectomy	7 (46.7)	10 (31.3)	
LAR/APR	3 (20.0)	10 (31.3)	

Data are presented as N (%) or mean ± SD

*ASA* American Society of Anesthesiologists, *BMI* body mass index, *LAR* low anterior resection, *APR* abdominoperineal resection

### Primary Outcome

The number of adequate and inadequate steps was compared between the two groups. Two comparisons were made: firstly, the video recordings of the study group versus the operative notes of the historic control group, and secondly, the video recordings and operative notes of the study cases combined versus the operative notes of the historic control cases (Table 2). Respectively, significant differences in favor of the study population were found regarding availability of information on the introduction of trocars under vision, overall exploration of the abdominal cavity, inspection of the liver, mobilization and resection, exploration of the resection specimen, and the accumulative steps. Information on vascular control was significantly more often available in the operative notes compared with the video recordings.

**Table 2:** Amount of adequate steps

	Study cases (*n* = 158)				Historic control (*n* = 343) adequate
	Video recording		Video recording and operative note combined		
	Adequate	*p* value	Adequate	*p* value	
Step 1 Introduction of trocars under vision	12 (80.0)	.004	14 (93.3)	<.001	10 (31.3)
Step 2 Exploration	35 (77.8)	.014	40 (88.9)	<.001	57 (60.6)
Inspection of the liver	14 (93.3)	<.001	14 (93.3)	<.001	10 (31.3)
Inspection of the tumor	8 (53.3)	.599	12 (80.0)	.182	17 (53.1)
Inspection of the peritoneum	13 (86.7)	.583	14 (93.3)	1.000	30 (93.8)
Step 3 Vascular control	8 (40.0)	.006	13 (65.0)	.371	32 (72.7)
Step 4 Mobilization and resection	24 (72.7)	<.001	24 (72.7)	<.001	26 (35.6)
Exploration of resection specimen	9 (60.0)	<.001	9 (60.0)	<.001	1 (3.1)
Identification of left ureter	11 (100)	.534	11 (100)	.534	18 (85.7)
Step 5 Creation of anastomosis	16 (53.3)	.376	23 (76.7)	.354	41 (60.3)
Anastomosis	10 (66.7)	.481	14 (93.3)	.406	25 (78.1)
Step 6 Closure	10 (66.7)	.753	12 (80.0)	.202	19 (59.4)
Total adequate steps	105 (66.5)	.024	126 (79.7)	<.001	185 (53.9)

Data are presented as N (%). *p* value obtained from comparison with historic control group

The average score for the amount of available information was 54.29 points (±15.42 SD) for the operative notes in the control group, 67.08 points (±13.81 SD) for the video recording alone, and 80.53 points (±11.72 SD) for the combination of video recording and operative note. Video recording alone and the combination of video recording and operative note scored significantly higher compared with the operative notes in the historic control group (*p* = .039; *p* = <.001, respectively).

When comparing the operative notes of the historic control cases to the operative notes of the study cases, the overall number of adequate steps were 185 (53.9 %) and 84 (53.2 %) (*p* = .648), respectively, and the average score for the amount of available information was 54.29 points (±15.42 SD) and 53.24 points (±13.26 SD) (*p* = .705) respectively.

### Secondary Outcome

Postoperative outcomes within 30 days after surgery are summarized in Table 3. Aside from a significant difference in the postoperative length of stay in favor of the study group (8.80 ± 9.01 vs. 10.44 ± 6.44 SD; *p* = .016), there was no significant difference found between the study cases and the historic control.

**Table 3:** Postoperative outcomes ≤30 days

Parameter	Study cases (*n* = 15)	Historic control (*n* = 32)	*p* value
Duration of surgery (minutes)	141.89 ± 79.14	143.84 ± 37.70	.257
Blood loss (ml)^a^	131.82 ± 78.34	104.69 ± 26.52	.284
Clavien-Dindo^b^			.292
Grade I	10 (66.7)	13 (40.6)	
Grade II	3 (20.0)	15 (46.9)	
Grade III	1 (6.7)	3 (9.4)	
Grade IV	1 (6.7)	1 (3.1)	
Surgical wound infection	1 (6.7)	4 (12.5)	.545
Intra-abdominal abscess	0 (0.0)	2 (6.3)	.322
Urinary tract infection	0 (0.0)	4 (12.5)	.152
Respiratory tract infection	1 (6.7)	6 (18.8)	.278
Cardiological complication	1 (6.7)	1 (3.1)	.575
Neurological complication	2 (13.3)	1 (3.1)	.182
Postoperative ileus	0 (0.0)	6 (18.8)	.073
Postoperative bleeding	0 (0.0)	1 (3.1)	1.000
Anastomotic leakage	2 (13.2)	1 (3.1)	.235
Postoperative length of stay (days)	8.80 ± 9.01	10.44 ± 6.44	.016

Data are presented as N (%) or mean ± SD

*DVT* deep venous thrombosis, *PE* pulmonary embolism

^a^Minimal blood loss set at ≤100 ml

^b^Postoperative morbidity and mortality according to Clavien-Dindo classification

## Discussion

Very few published studies analyzed the possible advantages of operative video recording prospectively. This pilot study is the first study evaluating operative video recording during colorectal cancer surgery. Our findings confirm the feasibility of systematic video registration in colorectal cancer surgery, as is shown in prior research.^17^ It also demonstrates the improved availability of essential information. The best results are obtained by combining video recording with the traditional operative note. This improvement might also be caused by the more stepwise approach during systematic video registration and the Hawthorne effect—improved performance due to the subject’s awareness that it is being recorded.

About half of the steps in the operative note were described in an adequate manner, which is in accordance with similar published findings in different fields of surgery.^18^–^21^ According to our results, adding systematic video recording to the traditional operative note would increase the total amount of available adequate information by almost 50 %. The most contributing steps to this increment are: introduction of trocars under vision, exploration of the abdomen, inspection of the surgical specimen, and, although not significant, the creation of the anastomosis. All these steps contain important information regarding either the procedure or further management of patient care. It is important to introduce the trocars under vision, because this otherwise poses risk to intra-abdominal injuries.^22^,^23^ The importance of an adequate inspection of the abdomen, including the liver and its surrounding ligaments, the parietal peritoneum, and the tumor, is to determine the operability of the patient and whether or not it is necessary to convert to open surgery. The surgical specimen should be inspected to make sure the tumor has been removed and the resection margin is sufficient. If flawed, the surgeon can then still act on these findings.

With regard to operative vascular control, a difference was found favoring the operative note over video. This is mainly due to atypical resections such as resection of the splenic flexure in which vascular control is at the level of peripheral vessels. Furthermore, especially in typical left-sided resections with central vascular control, it was sometimes difficult to assess the anatomy in the video without the explanation of the surgeon.

There was no significant difference found between the operative notes of the historic control group and those of the study cases in both the number of adequate steps and the average score for the amount of available information, which would suggest that participation of the study and the knowledge of the checklist by the surgeons do not bias the increase in available information when operative note and video registration are combined.

No significant difference was found in postoperative outcomes within 30 days, apart from postoperative length of stay. Because of the small sample size in the study group, this result should be considered thoughtfully.

Although video recording during laparoscopy has minimal impact on the surgical procedure, it might be considered impractical if recording could only be started and stopped outside the sterile area (e.g., on the laparoscopy tower, handled by an operating room assistant). This problem can be avoided by using a laparoscope with a dedicated recording button, or by using a recording remote inside the sterile environment, thus giving complete control to the surgeon’s team.

The video recordings in this study are fragments, aimed to capture the specific key moments in the surgical procedure instead of using a full-length recording. This results in a manageable amount of content and minimizes the required digital storage space. However, because of the fragmentation, it is sometimes difficult for reviewers to recognize certain structures in that particular fragment. A great improvement to this matter could be the addition of audio to the video recording, where the surgeon can verbally annotate the given procedure.

In addition to the improvement in available operative information and possibly surgical outcomes, video recording might also be useful for patient and family information and education and research purposes regarding effects of specific surgical techniques, as well as situational team awareness in the operating room. Also, it can result in improved communication between physicians from the treating team (e.g., surgeon and oncologist).^24^

In conclusion, peroperative systematic video registration in colorectal cancer surgery is feasible and early results regarding an increase in available intraoperative information are promising. An international multicenter study is currently being organized to evaluate the effect of video capture on surgical quality and patient outcomes.
